# The Validation of Automated Social Skills Training in Members of the General Population Over 4 Weeks: Comparative Study

**DOI:** 10.2196/44857

**Published:** 2023-04-27

**Authors:** Hiroki Tanaka, Takeshi Saga, Kota Iwauchi, Masato Honda, Tsubasa Morimoto, Yasuhiro Matsuda, Mitsuhiro Uratani, Kosuke Okazaki, Satoshi Nakamura

**Affiliations:** 1 Nara Institute of Science and Technology Ikoma Japan; 2 Department of Psychiatry Nara Medical University Kashihara Japan; 3 Osaka Psychiatric Medical Center Hirakata Japan

**Keywords:** social skills training, conversational agents, role-play, feedback, multimodal, long-term training effects

## Abstract

**Background:**

Social skills training by human trainers is a well-established method of teaching appropriate social and communication skills and strengthening social self-efficacy. Specifically, human social skills training is a fundamental approach to teaching and learning the rules of social interaction. However, it is cost-ineffective and offers low accessibility, since the number of professional trainers is limited. A conversational agent is a system that can communicate with a human being in a natural language. We proposed to overcome the limitations of current social skills training with conversational agents. Our system is capable of speech recognition, response selection, and speech synthesis and can also generate nonverbal behaviors. We developed a system that incorporated automated social skills training that completely adheres to the training model of Bellack et al through a conversational agent.

**Objective:**

This study aimed to validate the training effect of a conversational agent–based social skills training system in members of the general population during a 4-week training session. We compare 2 groups (with and without training) and hypothesize that the trained group’s social skills will improve. Furthermore, this study sought to clarify the effect size for future larger-scale evaluations, including a much larger group of different social pathological phenomena.

**Methods:**

For the experiment, 26 healthy Japanese participants were separated into 2 groups, where we hypothesized that group 1 (system trained) will make greater improvement than group 2 (nontrained). System training was done as a 4-week intervention where the participants visit the examination room every week. Each training session included social skills training with a conversational agent for 3 basic skills. We evaluated the training effect using questionnaires in pre- and posttraining evaluations. In addition to the questionnaires, we conducted a performance test that required the social cognition and expression of participants in new role-play scenarios. Blind ratings by third-party trainers were made by watching recorded role-play videos. A nonparametric Wilcoxson Rank Sum test was performed for each variable. Improvement between pre- and posttraining evaluations was used to compare the 2 groups. Moreover, we compared the statistical significance from the questionnaires and ratings between the 2 groups.

**Results:**

Of the 26 recruited participants, 18 completed this experiment: 9 in group 1 and 9 in group 2. Those in group 1 achieved significant improvement in generalized self-efficacy (*P*=.02; effect size *r*=0.53). We also found a significant decrease in state anxiety presence (*P*=.04; *r*=0.49), measured by the State-Trait Anxiety Inventory (STAI). For ratings by third-party trainers, speech clarity was significantly strengthened in group 1 (*P*=.03; *r*=0.30).

**Conclusions:**

Our findings reveal the usefulness of the automated social skills training after a 4-week training period. This study confirms a large effect size between groups on generalized self-efficacy, state anxiety presence, and speech clarity.

## Introduction

### Background

Social skills training (SST) has been widely adopted to help people who lack social and communication skills. It is used in hospitals, employment support facilities, workplaces, schools, and other institutions. A human trainer generally conducts SST to promote appropriate social and communication skills, strengthen the individual’s social self-efficacy, and reduce social anxiety [1,2]. The method of Bellack et al [3], or step-by-step SST, is a well-structured and widely used evidence-based approach inspired by the 5 core principles of social learning theory: modeling, shaping, reinforcement, overlearning, and generalization. This method defines the SST framework and its 4 basic skills: expressing positive feelings, listening to others, making requests, and declining requests. However, it is cost-ineffective because those who need to receive training must visit the place where the training is conducted (eg, hospitals and employment support facilities). Accessibility is further limited due to the low number of professional trainers, especially in rural areas. Our goal is to provide SST anywhere and anytime.

Other research groups, along with our efforts, have been conducting studies to automate SST using conversational agents [4,5] or robots [6], and these works have led to the development of automatic SST [7-12] through simulating human-led SST [13-15]. Among the various features of conversational agents, our SST system includes video modeling of human behavior, automatic real-time behavior recognition, and feedback. Our system’s specialty is to follow the procedure of human SST [1]. We also collected human SST data and integrated a social skills prediction model into the automatic system.

Several aspects of such a system have been evaluated in terms of training effect by short-term single-group intervention [13], appearance [14], and social self-efficacy [15]. However, the long-term effect of such an SST system and comparison with a control group have not been evaluated. Aside from SST, several studies have investigated the training effect of such mental health measures [16-18]. Single-group analysis was also performed in the context of social cognition training systems [19]. Much of this research used questionnaires, such as the Patient Health Questionnaire-9 [20], and emotion recognition tasks [21] to provide an evaluation scale. However, no consistent improvement or enhanced skills have been measured by questionnaires [16,19].

As a preliminary step, this study proposed to validate our SST system in members of the general population over 4 weeks. We separated the participants into 2 groups (with and without training) and clarified the training effect through evaluations before and after the 4-week training period. We hypothesized that the trained group would demonstrate a greater change in their social skills compared with the nontrained group.

### Automated SST

**Figure 1:**
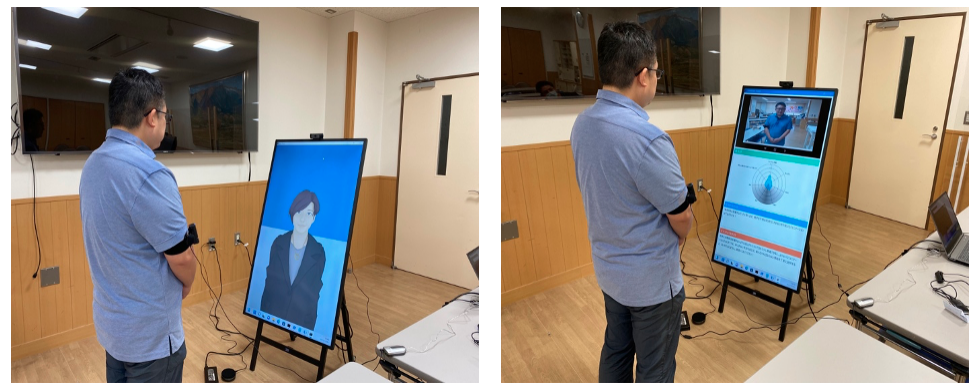
Social skills training system (left: role-playing; right: feedback).

We built a fully automated SST system using the Greta platform [4] and a conversational agent named Rei (Figure 1). Our system is capable of speech recognition, response selection, and speech synthesis and can also generate facial expressions, gestures, and head nods. Nonverbal behaviors are generated in the specific commands embedded in the dialogue responses. This system works in real time as a Windows application. The conversation agent’s appearance and sex can be changed. Previously, we designed anime-type female characters and investigated the acceptability and trustworthiness of their appearance [14]. This system is also applicable for use by healthy people, not only people with social disorders.

We created 4 tasks based on the basic SST model as well as scenarios for them: declining requests, listening to others, making requests, and expressing positive feelings. These selected tasks reflect the 4 basic tasks used in the Bellack method. Among these, declining a request is the most difficult [3]. When declining a request or listening to another person, the initiative is with the system that holds the floor. For the other 2 tasks, the initiative is on the learner’s side with the system in the responsive position.

After a brief greeting, the conversational agent explains to the participants the importance of the training task. The system records the users’ voices and images with a pin microphone and a webcam to assess user behavior. During role-play, the system perceives the user’s utterances by speech recognition and responds based on keywords (“yes,” “I understand,” “I have,” etc) prepared for it by rule-based interaction scenarios (Figure 1, left). We used Google Cloud Services for the speech recognition and speech synthesis modules. If the keywords were not directly included in the speech recognition output, we used the Bidirectional Encoder Representations from Transformers (BERT) model [22] to calculate the cosine distance to the above keywords at the sentence unit and chose the closest keywords. We created 7 role-play variations for each of the 4 tasks by referring to the SST data in 1-on-1 and 1-on-2 situations conducted by psychiatrists and people with autism spectrum disorders or schizophrenia, as well as healthy controls who were previously recorded [23]. The role-play variations included the following topics: hospital, home, school, workplace, and friends. In this study, for the selected scenarios, we excluded the hospital situation since our participants were from healthy populations.

We constructed a score evaluator from the role-play videos and automatically predicted 7 items: eye contact, body orientation, facial expression, vocal variation, clarity, fluency, and social validity. Each of these was rated by our psychiatrist evaluators on a 5-point scale. We predicted the ratings based on user behavioral indicators using multimodal features (Praat [24], OpenFace [25], and OpenPose [26]) and BERT [22] similarity scores between the utterances spoken by the conversational agent and the users. Random forest predicted the scores using these features, and 2 psychiatrists rated the ground truth of these values. We previously reported our detailed prediction performance and the correlation coefficient between the ground truth, and the predicted values was a maximum of 0.53 [27]. Depending on the evaluation results, a radar chart, positive comments, and corrective comments were presented on a screen with video clips, and the comments were read aloud by the conversational agent (Figure 1, right). The radar chart shows the evaluation values.

In SST, role-play rehearsals by participants are always immediately followed by positive feedback on what specifically a person did well. Here, a genuinely positive aspect must be found in even the poorest role-play performance. Our SST system praises the learner and provides positive reinforcement. The conversational agent and feedback are displayed through digital signage, and feedback is provided in full-screen view after the calculation is finished. The detailed SST system description can be found in a previous work [28].

## Methods

We followed the CONSORT (Consolidated Standards Of Reporting Trials) checklist to describe the method [29].

### Trial Design

We conducted a nonrandomized comparative study in members of a healthy population for a preliminary evaluation of the SST system. The allocation was not concealed for either the participants or the examiner. Participants could choose the group they wanted to register with depending on their availability for the number of visits to the examination room. The participants were not informed about the tasks of the other group. The trainers and third-party raters did not know the group participants. We conducted the study in July and August 2022, with July 23 being the pretraining evaluation date and August 28 being the posttraining evaluation date.

### Ethics Approval

This study was conducted in accordance with the Helsinki Declaration guidelines. Ethical approval was obtained from the Nara Institute of Science and Technology (Reference No. 2018-I-1), and we obtained informed consent from all participants.

### Participants

We asked a human resources company to advertise and recruit the participants. The eligibility criteria included (1) aged between 20 and 35 years and (2) a male and female balance of about 50%. We also attempted to balance the ages and sexes between the 2 groups. Exclusion criteria included (1) a history of epilepsy; (2) history of neurological disease; (3) history of substance abuse; (4) history of addiction; (5) history of head injury; (6) history of treatment at psychiatric hospitals; and (7) history of cataracts, amblyopia, strabismus, nystagmus, or ptosis. This exclusion criterion was originally designed to be used for future comparisons with people with autism spectrum disorders and schizophrenia.

In all, 13 Japanese participants were recruited for group 1 and 13 for group 2. Because half of the participants will wear eye trackers, they were asked not to wear colored eye contacts. Since this study was a preliminary step to test the system, we followed previous works regarding sample size [13,16,19]. We selected between 10 and 13 participants because this was a feasible number in terms of staff operation for a 1-day data collection of pre- and posttraining evaluations. We did not inform the participants of the SST system details in advance. They were also asked to come to both the pre- and posttraining evaluations. Group 2 was not trained by the system, so they were asked to come only to the pre- and posttraining evaluation sessions. The participants were paid an honorarium according to their group.

In all, 8 participants were ultimately unable to complete the experiment: 3 participants in group 1 and 3 participants in group 2 did not come to the pretraining evaluation; 1 participant in group 1 was unable to complete the training due to an isolation for COVID-19; and finally, 1 participant in group 2 did not come to the posttraining evaluation.

Two trainers and third-party raters, who are psychiatrists with SST experience, participated in our study.

### Interventions

We invited group 1 to visit Nara Institute of Science and Technology’s examination room every week (each Tuesday or Thursday) to use the system. Each training session consisted of SST with the conversational agent for 3 different skills from the basic 4 skills. The duration of the training per person was a maximum of 30 minutes. The sessions continued for 4 weeks, so participants attended 4 training sessions. None of the role-play scenarios were repeated in the 4-week training. The system was launched by the examiners (first or second authors) and worked automatically. One examiner (first or second author) remained in the same room with the participants to observe whether the system worked properly and safely. However, this potentially affected the intimacy between the participant and the conversational agent.

### Outcomes

Pre- and posttraining evaluations included the General Self-Efficacy Scale [30] (Japanese version [31]), Social Responsiveness Scale–2 [32] (Japanese version [33]), State-Trait Anxiety Inventory (STAI) [34] (Japanese version [35]), Liebowitz Social Anxiety Scale [36] (Japanese version [37]), and Kikuchi Scale of Social Skills–18 in Japanese [38]. We used Japanese versions of the questionnaires relevant to social skills, self-efficacy, and social anxiety. We obtained not only the total score but also the subscales for each questionnaire.

To evaluate the generalizability of social skills other than basic tasks, we also conducted 3 role-play scenarios that were not included in the basic tasks of Bellack et al [3] based on the performance test’s manual [39] that require social cognition and expression of the participants in new role-play scenarios. We conducted a practice session on starting a conversation and performing 3 role-plays for scenarios that require social cognition and expression: (1) understanding and expressing empathic behaviors, (2) self-disclosure, and (3) social problem-solving with one’s mother. We used Tobii Pro Glasses (version 3) to measure participants’ eye gaze during the role-play. Due to the limited availability of eye-tracker equipment, half of the participants in both groups wore a glass-type eye tracker.

Two psychiatrists with SST experience joined this study as role-playing interlocutors. We controlled the condition so that there was no difference in the trainers between groups and the evaluation stages. Due to COVID-19 concerns, a transparent partition was placed between the participants and trainers. A video camera was placed behind each conversationalist to record the other individual at chest level from the front.

The participants were asked to answer questions regarding social anxiety (no anxiety at all=1, somewhat anxious and a slight effect on performance=3, and strong anxiety and unable to perform=5) and social self-efficacy (good=1, neither=3, and bad=5) for the role-playing scenario after performing it. We also collected the evaluation data of the 2 interlocutor trainers by third-party evaluators. Since these ratings are almost all maximum, we omit an analysis of the trainers’ evaluations in this paper.

For the participant evaluation, we obtained third-party ratings using a Likert scale from 0 to 5 for eye contact, body direction and distance, facial expression, vocal variation, clarity, fluency, and social appropriateness [39]. For this evaluation, the third-party raters watched the recorded videos from the front-view, and the raters were not informed as to whether these were pre- or posttraining sessions or group 1 or group 2 (randomized by a computer).

Clarity evaluates how clearly and logically the participant is trying to express what they want to say. Since the required skills depend on each situation, the social appropriateness differs depending on each SST task. Let us explain examples of social appropriateness for the basic tasks of Bellack et al [3]. The task of listening to others, which determines whether the participants paid attention to the interlocutor, includes nodding, back-channel responses, and other nonverbal behaviors (eg, eye contact and smiling). For the task of expressing positive feelings, social appropriateness involves expressing attention to the interlocutor’s responses and the suitability of the participant’s speech content. For the task of expressing positive feelings, social appropriateness assesses whether they explained the details of their request, including what kind of help they need. It also includes whether they listened to the interlocutor. For the declining task, social appropriateness is concerned with whether they expressed contrition and appropriate reasons for their refusal. It also includes whether they proposed alternatives to the requests (eg, “I’m sorry but I propose to do it next time”), which is an important act for the situation.

After confirming areas of agreement, we calculated the averages of the 2 raters. For this study, we set the primary outcome as the third-party ratings and the secondary outcome as the other questionnaires.

### Statistical Analysis

We calculated the difference values between groups in terms of post- and pretraining evaluations. We used 1-tailed Wilcoxson Rank Sum tests while generally hypothesizing that group 1 would show larger improvement than group 2. We also reported the effect size *r* between groups. We analyzed the subscales of the questionnaires in addition to the total scores. To confirm the agreement of third-party ratings, we calculated the intraclass correlation coefficient using the 2-way random-effects model with a consistency-type analysis [40]. For the performance test on 3 role-plays, we concatenated the 3 role-play results to calculate the statistics since there was no significant difference between the role-play scenarios. For the statistical analysis, we used the R software (R Foundation for Statistical Computing) [41]. Specifically, we used the *stringr* and *irr* libraries and the Wilcoxson Rank Sum test function.

## Results

### User Statistics

Of the 26 recruits, 18 completed this study. Group 1 had 6 male and 3 female participants, and the mean age was 27.22 (SD 4.66) years. Group 2 had 5 male and 4 female participants, and the mean age was 27.33 (SD 4.74) years. There was no significant difference in terms of age (2-tailed Wilcoxson Rank Sum tests, *P*>.99) and sex (Fisher exact test, *P*>.99) between the groups.

### Evaluation Outcomes

Table 1 presents the results of the pretraining evaluation and the difference between the post- and pretraining evaluations from the questionnaires. Effect size *r* and *P* values are also reported in the pre-post values. Note that we did not confirm significant differences between the 2 groups at pretraining evaluation values (all *P*>.10). We can see that the General Self-Efficacy Scale was significantly improved in group 1 compared with group 2 (*P*=.02; *r*=0.53). We can also see that state anxiety presence was significantly weakened in group 1 compared with group 2 (*P*=.04; *r*=0.49). Regarding the performance test, the intraclass correlation coefficients between the 2 raters were as follows: eye contact=0.86, body direction and distance=0.80, facial expression=0.89, vocal variation=0.84, clarity=0.80, fluency=0.88, and social appropriateness=0.78 (all *P*<.001), which show good agreement. Results of the performance test showed significant changes in clarity in group 1 compared with group 2 (*P*=.03; *r*=0.30). Regarding the other scores, we found that some results were prone to significant features, but there were no significant differences between groups.

**Table 1:** Nonparametric analysis of rank sum test for differences between groups. Effect size r and *P* values correspond to post-pre values between the 2 groups.

		Pretraining		Posttraining			Posttraining-pretraining difference			Effect size *r*		*P* value
		Group 1 (n=9), mean (SD)	Group 2 (n=9), mean (SD)	Group 1 (n=9), mean (SD)	Group 2 (n=9), mean (SD)	Group 1 (n=9), mean (SD)		Group 2 (n=9), mean (SD)				
**General Self-Efficacy Scale**												
	Total	8.22 (3.96)	7.44 (4.77)	8.67 (3.61)	6.22 (4.55)	0.44 (3.97)		–1.22 (1.09)	0.42		.07^a^	
	Normalized score	2.44 (1.13)	2.67 (1.22)	2.89 (1.05)	2.22 (1.30)	0.44 (1.13)		–0.44 (0.53)	0.53		.02^b^	
**STAI^c^ (inversed^d^)**												
	Total (state)	42.00 (7.97)	41.89 (10.36)	39.67 (9.19)	43.89 (9.62)	–2.33 (5.17)		2.00 (6.89)	0.36		.12	
	State anxiety presence	44.44 (5.79)	43.00 (5.59)	42.56 (8.35)	45.22 (7.07)	–1.89 (4.48)		2.22 (6.26)	0.49		.04^b^	
	State anxiety absence	44.44 (7.94)	46.22 (12.40)	42.67 (6.93)	46.89 (9.25)	–1.78 (5.67)		0.67 (6.78)	0.16		.50	
	Total (trait)	47.33 (10.51)	49.00 (12.54)	46.56 (9.98)	49.78 (11.38)	–0.78 (9.71)		0.78 (5.38)	0.12		.62	
	Trait anxiety presence	51.67 (11.19)	52.00 (11.28)	49.78 (9.36)	51.78 (10.23)	–1.89 (10.68)		–0.22 (5.04)	0.13		.59	
	Trait anxiety absence	40.67 (8.58)	43.89 (11.56)	41.78 (8.47)	45.89 (10.06)	1.11 (7.10)		2.00 (4.80)	0.16		.50	
**LSAS^e^ (inversed)**												
	Total	49.78 (26.47)	46.22 (30.76)	56.00 (31.14)	51.11 (33.14)	6.22 (29.28)		4.89 (9.99)	0.15		.52	
	Total (anxiety)	24.00 (13.02)	27.00 (14.73)	27.44 (14.73)	29.33 (18.81)	3.44 (17.05)		2.33 (6.48)	0.09		.71	
	Performance anxiety	11.67 (6.52)	11.67 (9.77)	13.89 (7.01)	13.56 (10.19)	2.22 (8.64)		1.89 (2.42)	0.19		.43	
	Social anxiety^f^	12.33 (6.76)	15.33 (9.29)	13.56 (8.23)	15.78 (9.32)	1.22 (8.66)		0.44 (5.36)	0.05		.82	
	Total (avoidance)	25.78 (16.02)	19.22 (13.75)	28.33 (17.30)	21.78 (15.59)	2.56 (13.25)		2.56 (6.02)	0.13		.58	
	Performance avoidance	11.78 (8.26)	8.44 (7.13)	13.44 (7.63)	9.78 (7.81)	1.67 (6.36)		1.33 (3.54)	0.15		.53	
	Social avoidance	14.00 (7.98)	10.78 (7.07)	14.89 (9.84)	12.00 (8.03)	0.89 (7.59)		1.22 (2.86)	0.14		.55	
**SRS-2^g^ (inversed)**												
	Total	69.78 (22.73)	67.89 (36.14)	70.78 (24.55)	67.67 (34.06)	1.00 (15.38)		–0.22 (7.93)	0.11		.65	
	Awareness	8.56 (3.43)	7.67 (4.21)	9.00 (2.92)	7.56 (3.75)	0.44 (0.51)		–0.11 (1.83)	0.06		.80	
	Cognition	12.89 (4.94)	14.22 (6.14)	11.89 (5.13)	13.22 (6.04)	–1.00 (4.33)		–1.00 (2.18)	0.10		.67	
	Communication	21.67 (9.49)	21.33 (14.06)	21.33 (8.62)	20.22 (12.25)	–0.33 (6.06)		–1.11 (3.92)	0.08		.73	
	Motivation	12.22 (3.56)	13.56 (7.09)	14.67 (4.87)	15.89 (7.46)	2.44 (5.83)		2.33 (3.24)	0.28		.22	
	Repetitive and restricted behaviors	14.44 (7.40)	11.11 (7.06)	13.89 (7.24)	10.78 (6.20)	–0.56 (4.13)		–0.33 (3.12)	0.11		.64	
**KiSS-18^h^**												
	Total	59.89 (12.33)	60.00 (16.40)	61.44 (11.50)	61.22 (12.84)	1.56 (6.65)		1.22 (5.70)	0.17		.48	
	Basic skills	9.22 (3.27)	9.44 (2.79)	9.89 (3.22)	10.22 (2.82)	0.67 (1.12)		0.78 (0.83)	0.11		.65	
	Advanced skills	10.56 (2.40)	10.67 (2.69)	10.78 (2.49)	11.11 (2.80)	0.22 (2.39)		0.44 (1.01)	0.09		.71	
	Emotion processing	9.33 (2.83)	9.56 (4.03)	9.67 (2.60)	9.44 (1.88)	0.33 (1.80)		–0.11 (2.26)	0.26		.27	
	Offensive	9.22 (2.33)	8.56 (3.28)	9.67 (2.24)	9.33 (2.40)	0.44 (1.67)		0.78 (1.92)	0.10		.66	
	Stress coping	10.11 (2.71)	10.11 (3.37)	9.78 (2.68)	10.00 (3.20)	–0.33 (1.73)		–0.11 (2.09)	0.15		.52	
	Planning	11.44 (1.81)	11.67 (1.09)	11.67 (1.87)	11.11 (2.26)	0.22 (1.09)		–0.56 (1.33)	0.41		.08^a^	
**Performance test^i^**												
	Anxiety (inversed)	2.22 (0.97)	2.25 (0.98)	1.93 (0.73)	2.26 (0.76)	–0.30 (0.99)		0 (0.73)	0.23		.08^a^	
	Self-efficacy^f^	2.56 (1.09)	2.44 (0.93)	2.33 (0.88)	2.44 (0.85)	–0.22 (0.75)		0 (0.78)	0.03		.84	
	Eye contact	4.35 (1.08)	4.52 (0.69)	4.37 (1.09)	4.48 (0.75)	0.02 (0.35)		–0.04 (0.40)	0.17		.21	
	Body direction and distance	4.44 (0.92)	4.61 (0.64)	4.52 (0.80)	4.56 (0.67)	0.07 (0.38)		–0.06 (0.35)	0.22		.10	
	Facial expression	3.96 (1.10)	4.02 (0.90)	4.04 (0.96)	4.07 (0.89)	0.07 (0.58)		0.06 (0.42)	0.10		.46	
	Vocal variation	4.26 (1.10)	4.48 (0.67)	4.37 (0.93)	4.57 (0.74)	0.11 (0.47)		0.09 (0.37)	0.07		.61	
	Clarity	4.17 (1.02)	4.46 (0.72)	4.48 (0.86)	4.46 (0.82)	0.31 (0.57)		0 (0.54)	0.30		.03^b^	
	Fluency	4.11 (1.11)	4.39 (0.68)	4.37 (0.96)	4.54 (0.76)	0.26 (0.67)		0.15 (0.48)	0.14		.30	
	Social appropriateness	4.11 (1.11)	4.39 (0.68)	4.37 (0.96)	4.54 (0.76)	0.26 (0.67)		0.15 (0.48)	0.14		.29	

^a^*P*<.10.

^b^*P*<.05.

^c^STAI: State-Trait Anxiety Inventory.

^d^Inversed indicates that greater values show lesser skills and higher anxiety.

^e^LSAS: Liebowitz Social Anxiety Scale.

^f^Note that self-efficacy and social anxiety were obtained from 3 role-plays.

^g^SRS-2: Social Responsiveness Scale–2.

^h^KiSS-18: Kikuchi Scale of Social Skills–18.

^i^The performance test was conducted in a concatenation of 3 role-play scenarios.

## Discussion

### Principal Results

Principal results include group 1 showing significant improvement in social skills (speech clarity). Our findings demonstrate the usefulness of the study in a 4-week training program. Our study confirmed the large effect size of the different groups. General self-efficacy improved and state anxiety presence was reduced because successful SST basically improves participant self-efficacy and lowers anxiety.

### Comparison With Prior Work

This is the first prototype of an SST system based on human SST. Aside from SST, several other works have investigated the training effect of such mental health measures [16-18]. Single-group analysis has also been performed in the context of social cognition training [19]. Most of this work used questionnaires and performance tests [20,21]. However, there was no consistent improvement or enhancement of skills, as observed through this work’s questionnaires. Additionally, there is no consistent questionnaire and evaluation criteria for SST in past studies, which makes it difficult to compare with this study. Previous evaluations include learning new social skills; improving assertiveness; hospital discharges; relapse rates; and effects on stress reduction, quality of life, symptoms, and hospitalization [3].

Our research demonstrates the system’s usefulness in achieving a training effect during 4 weeks of training and confirms a large effect size between groups for generalized self-efficacy, state anxiety presence, and speech clarity (*r*>0.30). Human trainers generally conduct SST to promote appropriate social and communication skills, strengthen an individual’s social self-efficacy, and reduce social anxiety [1,2].

Our SST system praises the learner and provides positive reinforcement, affecting gains in self-efficacy [15,42]. It can be argued that our SST strengthens self-efficacy, leading to behavioral modification [43]. We found a reduction of state anxiety presence and anxiety for role-playing since role-playing with the conversational agent is safe and provides successful user experiences to reduce anxiety. Another study also found that if encouragement and success experiences are present, the user experiences an increased sense of trust, security, and safety and a reduction in tension, threat, and anxiety [44]. Our primary outcome found improved speech clarity. Our SST system includes feedback with a concrete suggestion of behaviors, for instance, “You should provide more concrete reasons for declining.” This suggestion also leads to improvement in speech clarity. Previous work also reported an improved frequency of positive social behavior by human SST [45]. Heinssen et al [46] reported that participants learned, retained, and generalized new social skills through human SST.

In the future, the system needs to become more sophisticated in terms of modeling behaviors and the agent’s naturalistic nonverbal behaviors, including dyad synchronization in turn-taking or theory of mind [47,48].

### Limitations

This study is limited in its number of participants, which we need to increase in our future work to investigate a broader range of participants, including those with autism spectrum disorders and schizophrenia. This study was not a randomized controlled trial because participants were permitted to choose which group they wanted to register with. We found that some scores of participants in group 2 moved in the opposite direction. The negative effect of the first contact with the evaluation task on many social variables might have influenced participants, especially those in group 2, and the positive effects of training may be overestimated. Moreover, our participants are from healthy populations, so the baseline scores at the pretraining evaluation were already high. We also did not find a significant difference between trait-related measures, for example, trait anxiety and the Social Responsiveness Scale. A follow-up evaluation will also be needed to observe skills generalization and transformation over a long-term period. Furthermore, this study did not evaluate in terms of emotion recognition tasks [21]. By observing the history of the system’s development, we confirmed that some participants’ scores did not change between the beginning and end of the training, which might be related to the boredom of repetitive training. We should clarify the optimal training duration and number of role-play repetitions in the future. In addition, we should compare the SST system with human SST in terms of training effects in adults and children [49].

### Conclusion

This study validated an SST system using a 4-week training program. The trained group demonstrated significantly improved self-efficacy and reduced anxiety. Furthermore, we confirmed the large effect size in terms of speech clarity. We plan to extend this study to include people with autism spectrum disorders and schizophrenia, and the system needs to be further elaborated. It may also be impactful to a much larger group with different social pathological phenomena. We are now developing a web-based SST system that could be used as a supplement to human SST.

## Abbreviations

BERT: Bidirectional Encoder Representations from Transformers
CONSORT: Consolidated Standards Of Reporting Trials
SST: social skills training
STAI: State-Trait Anxiety Inventory
